# A new microscopy pipeline for studying the initial stages of nuclear and micronuclear rupture and repair

**DOI:** 10.3389/fcell.2024.1475095

**Published:** 2024-09-18

**Authors:** Melody Di Bona, Samuel F. Bakhoum

**Affiliations:** ^1^ Radiation Oncology, Memorial Sloan Kettering Cancer Center, New York, NY, United States; ^2^ Human Oncology and Pathogenesis Program, Memorial Sloan Kettering Cancer Center, New York, NY, United States

**Keywords:** high-resolution microscopy, micronuclear rupture, nuclear envelope rupture, nuclear envelope repair, real-time protein recruitment analysis, spatiotemporal protein recruitment analysis, micronuclear repair impairment

## Abstract

Nuclear envelope repair is a fundamental cellular response to stress, especially for cells experiencing frequent nuclear ruptures, such as cancer cells. Moreover, for chromosomally unstable cancer cells, characterized by the presence of micronuclei, the irreversible rupture of these structures constitutes a fundamental step toward cancer progression and therapy resistance. For these reasons, the study of nuclear envelope rupture and repair is of paramount importance. Nonetheless, due to the constraint imposed by the stochastic nature of rupture events, a precise characterization of the initial stage of nuclear repair remains elusive. In this study, we overcame this limitation by developing a new imaging pipeline that deterministically induces rupture while simultaneously imaging fluorescently tagged repair proteins. We provide a detailed step-by-step protocol to implement this method on any confocal microscope and applied it to study the major nuclear repair protein, barrier-to-autointegration factor (BAF). As a proof of principle, we demonstrated two different downstream analysis methods and showed how BAF is differentially recruited at sites of primary and micronuclear rupture. Additionally, we applied this method to study the recruitment at primary nuclei of the inner nuclear membrane protein LEM-domain 2 (LEMD2) and Charged Multivesicular Protein 7 (CHMP7), the scaffolding protein of the endosomal sorting complex required for transport III (ESCRT-III) membrane remodeling complex. The CHMP7-LEMD2 binding is the fundamental step allowing the recruitment of ESCRT-III, which represents the other major nuclear repair mechanism. This demonstrates the method’s applicability for investigating protein dynamics at sites of nuclear and micronuclear envelope rupture and paves the way to more time-resolved studies of nuclear envelope repair.

## 1 Introduction

Nuclear envelope rupture at primary nuclei is a recurring event in cells undergoing shear or confinement stress (Maciejowski and Hatch, 2020), and as such, repair mechanisms have evolved to restore nuclear compartmentalization, fundamental for cell survival (Denais et al., 2016a; Halfmann et al., 2019). This is especially true in cancer (Denais et al., 2016b; Vargas et al., 2012), where mechanisms of nuclear envelope repair such as barrier-to-autointegration factor (BAF) (Halfmann and Roux, 2021) and the membrane remodeling complex ESCRT-III (Raab et al., 2016; Olmos et al., 2015) can influence patient prognosis (Li et al., 2018; Sears and Roux, 2020). Moreover, most aggressive human tumors are characterized by chromosomal instability (Bakhoum and Cantley, 2018), a hallmark of cancer often associated with poor prognosis, distant metastasis, and therapy resistance (Al-Rawi et al., 2024; Ippolito et al., 2021; Chen et al., 2024). Chromosomally unstable cells harbor aberrant structures called micronuclei. Micronuclei experience rupture far more frequently than primary nuclei. Moreover, micronuclear rupture is generally irreversible (Hatch et al., 2013; Vietri et al., 2020), suggesting a wide dysregulation of repair mechanisms (Martin et al., 2024). Thus, micronuclear collapse represents a milestone in cancer progression and aggressiveness (Di Bona and Bakhoum, 2024).

For these reasons, both primary nuclei and micronuclei have been the subject of numerous studies seeking to elucidate the dynamics of protein recruitment in the context of membrane repair and DNA sensing (Vietri et al., 2020; Martin et al., 2024; Willan et al., 2019; Tang et al., 2022; Mohr et al., 2021; Mackenzie et al., 2017). To date, such investigations have primarily employed spatially resolved techniques such as confocal and high-resolution microscopy. However, when the temporal dimension is integrated, both temporal and spatial resolutions are compromised. The stochastic nature of the rupture event necessitates prolonged live-cell imaging experiments, introducing challenges in balancing imaging file size and mitigating phototoxicity, while maintaining adequate temporal resolution (Ettinger and Wittmann, 2014). Moreover, the extended measurement duration imposes a constraint to the achievable spatial resolution as it necessitates to account for both cellular and intracellular movements (Liu et al., 2018). Finally, this approach is inherently inefficient as the molecular events occurring post-rupture generally take place within a time window shorter than the interval between consecutive frames.

In this study, we introduce a novel high-resolution fluorescence microscopy-based technique that addresses these challenges by enabling timely and precise recording of protein recruitment at the specific site of rupture. To this end, we devised a pipeline that induces rupture in both micronuclei and primary nuclei by exposing the nuclear envelope to a high-intensity 405-nm laser (Halfmann et al., 2019; Denais et al., 2016b; Raab et al., 2016; Kono et al., 2022; Penfield et al., 2018) while imaging. This pipeline allows for the recording of a fluorescence time-lapse with temporal resolution adjustable down to seconds, depending on the timescale of the event being studied. First, we characterized the best parameters for inducing both primary and micronuclear envelope rupture, identified with loss of compartmentalization through a fluorescent protein fused with a nuclear localization sequence (NLS-RFP) (Mackenzie et al., 2017; Hatch and Hetzer, 2016), while minimizing bleaching and phototoxicity. After validating the effective rupture of micronuclei via recruitment of the DNA sensing cyclic GMP-AMP synthase (cGAS) (Mackenzie et al., 2017), we applied this pipeline to study the recruitment of the nuclear envelope repair protein BAF at the site of micronuclear and primary nuclear rupture. Moreover, we demonstrated its applicability to study sequential protein recruitment, using as a proof of principle the recruitment of the ESCRT-III complex scaffold protein, CHMP7, through its interaction with the inner nuclear membrane protein LEMD2 (Gu et al., 2017).

We present a point-by-point protocol that can be applied to any confocal microscope or any microscope with a bleaching/FRAP module, followed by two different analysis applications, one with the open-source software ImageJ (Rasband, W.S., ImageJ, U.S. National Institutes of Health, https://imagej.nih.gov) and one with the 3D rendering software Imaris (Oxford Instruments).

## 2 Materials and equipment

• Adherent cells (we used HeLa wild-type, gift from the Cheeseman lab, Massachusetts Institute of Technology, purchasable from the ATCC)
• Lentiviral vectors or viral preparations for fluorescent protein expression (see Table 1)
• DMEM + high glucose + pen/strep + sodium pyruvate (MSKCC core facilities)
• Fetal bovine serum (Fisher Scientific, MT35015CV)
• Phosphate-buffered saline (MSKCC core facilities)
• Trypsin-EDTA (Fisher Scientific, 25-300-120)
• Blasticidin 10 mg/mL (InvivoGen, ant-bl-1)
• Puromycin 10 mg/mL (InvivoGen, ant-pr-1)
• Ibidi 8-well chambers (ibiTreat 90 u-slide, 80826-90, Ibidi)
• Live Cell Imaging solution (A59688DJ, Invitrogen)
• Hoechst 33342 (62249, Thermo Fisher)
• Optical fluorescence microscope with a minimum resolution of 250 nm. We recommend the use of a confocal microscope; the wide-field system could work if coupled with an FRAP/bleaching module. The microscope should be equipped with a temperature- and humidity-controlled chamber for live cell imaging. Here, we used an inverted Zeiss LSM880 (Carl Zeiss Microscopy GmbH, Germany) equipped with an Airyscan detector (gain 850, digital gain 1) in the super-resolution mode. For image acquisition, we suggest using an objective with an optical magnification of at least ×60, oil or water immersion. In this study, we used a ×63 oil immersion Plan-Apochromat 63×/1.4 Oil DIC M27.
• For image processing, we suggest using deconvolution or other noise reduction algorithms with the microscope built-in software. Here, we processed the images using Airyscan processing in Zeiss ZEN black software using default parameters. For further image analysis, we show possible applications with the open-source software ImageJ (Rasband, W.S., ImageJ, U.S. National Institutes of Health, https://imagej.nih.gov) and the 3D reconstruction software Imaris (Oxford Instruments).
• For statistical analysis and graphical representation, we used the software GraphPad Prism 9 (https://www.graphpad.com/). Sketches were made using BioRender App (https://app.biorender.com; with publication license). Figures were prepared using Adobe Illustrator Software (Adobe).

**TABLE 1:** Lentiviral vectors for fluorescent protein expression.

Plasmid name	Protein of interest	Antibiotic selection	Origin	Catalog number
pVLX_puro-mCherry-NLS-TagRFP	NLS-RFP	Puromycin	Hatch lab	NA
cGAS-GFP	cGAS fused to EGFP	Puromycin	Maciejowski Lab	NA
EGFP-BAF	BAF fused to EGFP	Blasticidin	Addgene	101772
pGenLenti-CHMP7GFP	CHMP7-GFP	Puromycin	Insert from pMGF182,Addgene	plasmid #97006
pGenLenti-LEMD2mCherry	LEMD2-mCherry	Blasticidin	Insert from pMGF196,Addgene	Plasmid #97005

## 3 Methods

### 3.1 Generating cell lines

#### 3.1.1 Timing: at least 1 month before the experiment

Generate stable cell lines, rather than rely on transient transfection, expressing the proteins of interest fused with a fluorescent protein. Here, we used lentiviral vectors to generate stable HeLa cells expressing the proteins of interest. To produce the lentiviral vectors, we co-transfected the parental cell line HEK293 (ATCC, CRL-3216) with packaging vectors and the vector of interest listed in Table 1. We used Lipofectamine 2000 (Thermo Fisher Scientific 11668019), following the manufacturers’ instructions. We then collected the supernatant containing the lentiviral particles after 48 h and used it to transduce HeLa cells. For HeLa cell infection, we plated 200,000 cells resuspended in 1 mL of fresh media, together with 6 μL of polybrene (Santa Cruz Biotechnologies sc-134220) and 1 mL of virus suspension in a 6 well-plate. Cells were left to adhere with the virus for 24 h before changing the medium. Appropriate antibiotic selection was added after 48 h of infection. For double protein expression, we proceeded sequentially. We suggest sorting cells for fluorescent protein expression using fluorescence-activated cell sorting in addition to antibiotic selection. Selection of clones is recommended to ensure consistency.

### 3.2 Culturing cell lines

#### 3.2.1 Timing: 10 days before the experiment

Thaw the cell line expressing the chosen fluorescent protein/s at least 10 days before the experiment, and culture in DMEM high glucose supplemented with 10% FBS and with the appropriate antibiotic (5–20 μg/mL puromycin or blasticidin) and seed them in an appropriate container (1 × 10^6^ cells in a 10-cm dish). Passage the cells every 3–4 days for HeLa, or adjust depending on the cell line doubling time.

#### 3.2.2 Timing: the day before the experiment

Wash the cells with PBS, trypsinize them, and seed them at a density of 0.5–15 × 10^5^ cells in each of the four central wells of an 8-well ibidi chamber, for a maximum volume of 350 μL per well. Fill the border wells of the chamber with PBS to prevent evaporation of the media.

##### 3.2.2.1 STOP point

It is possible to plate the cells at a lower concentration and use them up to 3 days after plating them.

### 3.3 Staining adherent cells (optional, for DNA visualization)

#### 3.3.1 Timing: 1–2 h before the experiment

Wash away the media from the wells of the ibidi chamber, wash once with PBS, and incubate the cells for 15 min in a 37°C incubator containing a sterile solution of 2.5 μg/mL Hoechst 33342 dissolved in live cell imaging solution. When the incubation time is over, wash thoroughly 4x with live cell imaging and then replace with fresh live cell imaging solution.

Optional: if treatments are needed, depending on the duration of the treatment, change the media on the cells with the appropriate drug dissolved in the live cell imaging solution. If the treatment needs to be washed out, perform it before staining the cells.

### 3.4 Image acquisition

#### 3.4.1 Timing: 1 h before starting the experiment

Optional: perform any treatment longer than 1 h or that needs to be washed out, before setting the ibidi chamber in the microscope. For shorter treatments, we suggest changing the media with the live cell imaging solution mixed with the drug of interest from the ibidi well when the chamber is already in the incubation chamber of the microscope. This will minimize the time spent to allow the cells to settle.

1. Place the ibidi with the live cell imaging solution on the microscope, in the temperature-controlled chamber. Let the cells sit for at least 1 h to bring the glass and the oil of the objective, to temperature and to minimize temperature-driven objective and sample drift.
2. Set the optical configuration of the microscope, and turn on the appropriate lasers (intensity will depend on the fluorescent protein expression, but needs to be kept constant among conditions). We used a 488-nm laser line to image GFP-tagged proteins and a 561-nm laser line to image RFP- or mCherry-tagged proteins, both at 1.5% power. When imaging nucleic acid through Hoechst staining, we used a 0.7% power 405-nm laser line.

We used a pixel size of 30 nm, 512 × 512 pixels per image (13.5 μm × 13.5 μm). The regular imaging acquisition speed was 0.85 μs, with a scan time of 521 ms. We acquired one Z-slice every 500 nm for a total Z-stack of 3 μm for the primary nucleus and a Z-stack comprising the whole micronucleus (depending on micronuclear size). For the time lapse measurement, we acquired one Z-stack every 30 s, but depending on the speed of acquisition and on the expected time of the event under study, this time can be decreased to 10 s.

For the rupture, we selected a region of interest (ROI) of 12 × 18 pixels in a peripheral region of the micronucleus or primary nucleus and used a pixel dwell time (the time the laser is spending on each pixel) of 32.77 μs After the first frame of regular imaging, we bleached the ROI with a 405-nm laser at 95% and 100 iterations on the ROI before resuming regular imaging.

#### 3.4.2 Starting the experiment

3. Find the focus of the microscope on the adherent cells by using the oculars and an epifluorescence lamp or a built-in autofocus system.

##### 3.4.2.1 Optional: run a rupture test (requires NLS-RFP expression or other rupture marker)

4. Choose a cell with a good NLS-RFP expression and an intact micronucleus.
5. Define the Z-stack: choose the first and last plane of the series needed to be imaged. We suggest choosing a Z-stack that comprises the whole micronucleus.
6. Choose an ROI that is situated on the nuclear periphery or on the micronucleus. In systems allowing bleaching of a specific z-position, choose the central plane of the Z-stack as the bleaching plane. This will account for the intrinsic mobility of the micronucleus and ensure that the rupture site will be in focus during the entire measurement.
7. As a starting condition, use 100 iterations of the laser with a 32.77 μs pixel dwell time for bleaching. In experimental systems in which these bleaching conditions are not ensuring 100% rupture, the pixel dwell time or iterations can be increased until complete rupture is achieved.
8. Acquire the measurement, stopping after three frames after rupture. For frame sizes and intervals, refer to step 2. Assess the integrity of the micronucleus through NLS-RFP visualization (Supplementary Figure S2). Another possibility, although not tested here, is to measure the presence of NES-GFP.
9. Repeat steps 4–8 for five times, using a different cell each time.
10. If any of the micronuclei did not rupture, increase the pixel dwell time or the iterations, and repeat steps 4–8 for five times. Increase the conditions until all of the micronuclei rupture.

##### 3.4.2.2 Run a pilot measurement

11. Choose a cell with a good expression of the fluorescent protein under examination. For micronuclei studies, choose a cell with an intact micronucleus that is preferably not above the primary nucleus to maximize the precision of focus and imaging.
12. Define the Z-stack: choose the first and last plane of the series that needs to be imaged. We suggest choosing a Z-stack that comprises the whole micronucleus, while for primary nuclei imaging, we suggest a Z-stack of approximately 2–2.5 μm total.
13. Choose an ROI that is situated on the nuclear periphery or on the micronucleus. If your system allows it, choose a different Z-position for the bleaching and pick the central plane of the Z-stack (see step 6).
14. Start the measurement by acquiring as a starting point a time-lapse every 30 s.
15. If you notice that the protein of interest is already present in the first frame after rupture, then scale down the sampling time accordingly.

##### 3.4.2.3 Run the experiment

16. Repeat the steps 11–14 using the sampling time found in step 15, on a different cell. We recommend using different cells for different measurements to avoid the possibility that protein recruitment at one cellular site might affect recruitment time at a second site.
17. Start the measurement acquiring the time-lapse with the sampling time chosen previously. We recommend to not exceed a length of 1 h for the measurements to avoid bleaching, phototoxicity, and sample drifting.
18. Save the measurement file.
19. Optional: process the raw data (e.g., Airyscan processing, denoising, and deconvolution) and save the processed version.

##### 3.4.2.4 STOP point

20. Proceed with image analysis.

### 3.5 Analysis with Fiji

Once the live-cell measurements have been acquired, processed (in our case through the Airyscan processing function in Zeiss built-in software ZEN), and saved in native format (.czi), it is possible to proceed with the analysis. As an application example, we quantified the time of post-rupture recruitment for each protein of interest (POI) using the free image analysis software ImageJ, downloadable at (https://fiji.sc/). To enable the drag and drop option and the possibility to open microscope native formats in ImageJ, the BioFormats Importer plugin needs to be installed (downloadable through ImageJ-Plugins-BioFormats). A Z-projection using the maximum intensity is recommended (Image-Stacks-Z projects), but other Z-projection modes might be preferred by the user (e.g., mean intensity), depending on the application. Once a Z-projected file is generated, we recommend adjusting brightness and contrast (Image-Adjust-Brightness/Contrast) for each channel using the Auto function. At this point, the POI intensity can be manually calculated. We recommend selecting a round ROI (using the button “Oval selection”) on the background, on the POI at the beginning of the experiment, and on the POI after rupture. From this ROI, fluorescence intensity information is extracted. After confirming that the box of “Mean gray intensity” under the measurements settings is checked (Analyze-Set measurement), clicking Measure (Analyze-Measure) will return the mean intensity value of Background, POI at the beginning of the experiment, and POI at the selected frames after rupture. The post-rupture frame in which the POI appears at least 10 times brighter than the first frame (after background subtraction) is identified as the recruitment time. The precise time information is obtained from the raw data (Image-Show Info) and can then be used for further analysis or graphical representation.

### 3.6 Analysis with Imaris

As a second application example, we quantified the change of POI amount (volume of protein) over time after the rupture occurs. To do so, we used the 3D rendering software Imaris (we recommend using the 8.4 version or above). First, we converted the microscopy file in the native Imaris format. After opening it in “Slice View,” we created a “New Surface” corresponding to the POI.

To create the surface, we used an ROI that comprises the site of rupture for all the time frames. Then, we chose the channel corresponding to the POI, and, after smoothing using a surface detail of 0.0318 μm, we selected an automatic threshold. We then finalized the surface generation using the “track surface” option to allow for graphing the volume of the POI over time. After checking that the surface (i.e., the 3D-rendered POI) was correctly recognized on all the frames, we joined the tracks and the identified POI in one single surface tracked along the whole measurement (“edit Track” and “edit Surfaces”). We then selected the “Statistics” tab and generated a graph of the POI volume (other valuable measurement possibilities are area, volume overlap between different surfaces, or sphericity) over time. We finally exported these data in .csv files for further analysis or graphical representation.

## 4 Results

### 4.1 Nuclear and micronuclear rupture

Recognizing the need to understand nuclear envelope rupture and repair dynamics with high temporal resolution, we aimed to overcome the existing limitations posed by the inherent stochasticity of rupture and the lack of real-time visualization techniques with appropriate temporal resolution. Our goal was to develop a method that enables the study of protein recruitment at the rupture site during the critical initial minutes following rupture.

**FIGURE 1:**
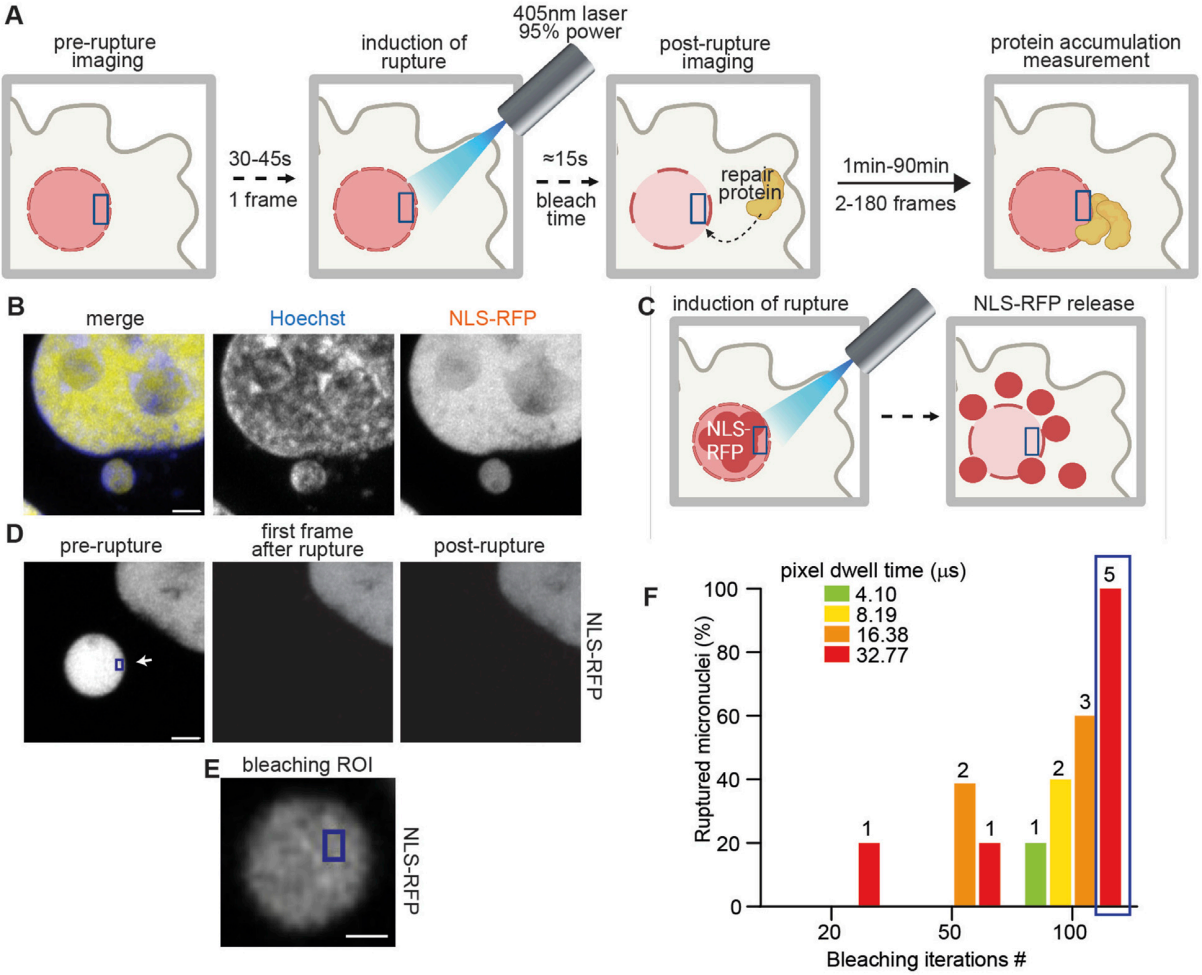
Laser-induced micronuclear rupture. **(A)** Schematic representation of the method workflow. An ROI is selected in the micronucleus or primary nucleus that, after the first frame of imaging, is exposed to a 405-nm laser at 95% power for 15 s. Imaging is then performed for the chosen time to record the behavior of repair proteins at the site of rupture. **(B)** Representative images of HeLa cells expressing NLS-RFP (yellow) and stained with Hoechst (blue). Scale bar = 2 μm **(C)** Schematic representation of NLS-RFP release in the cytosol after laser-induced rupture. **(D)** Representative images of a micronucleus in HeLa cells expressing NLS-RFP pre and post laser-induced rupture. Scale bar = 2 μm. The ROI used for bleaching (blue) is indicated with the white arrow. **(E)** Representative image of an ROI (blue) on a micronucleus in HeLa cells expressing NLS-RFP. Scale bar = 0.5 μm. **(F)** Graphical representation of micronuclear rupture efficiency after bleaching the ROI as in **(E)** using the indicated pixel dwell times and number of iterations. The number of ruptured micronuclei is indicated on the columns, and the total micronuclei probed for each measurement was 5. The blue box highlights the optimal conditions (enabling virtual 100% micronuclear rupture) that we chose for further bleaching.

To this aim, we optimized a microscopy pipeline that, by harnessing the ability to induce nuclear envelope rupture with a 405-nm laser (Denais et al., 2016b; Raab et al., 2016; Kono et al., 2022; Penfield et al., 2018), removes the stochastic variable and allows for proper time resolution in the moments immediately following this event (Figure 1A).

First, we sought to understand the optimal parameters to induce virtually 100% rupture events, while minimizing the exposure time to the 95% 405-nm laser light used to induce rupture. To discriminate between micronuclei ruptured or intact, we used HeLa cells expressing NLS-RFP, which is released into the cytoplasm upon loss of compartmentalization (Figures 1B–D; Supplementary Figure S1A). As a further validation, we used HeLa cells expressing NLS-RFP and cGAS-GFP, a DNA-recognizing protein that is rapidly recruited in bright foci at the site of primary or micronuclear rupture (Mackenzie et al., 2017; Agustinus et al., 2023) (Supplementary Figures S1B–D) We opted to maintain a consistent ROI size of 360 nm × 540 nm across all measurements (Figure 1E), as this was the minimum size that accommodated even the smallest observed micronuclei (data not shown). Thus, we characterized the probability of rupture while changing the pixel dwell time and the iterations on the ROI of the 405-nm laser. For the following applications, we used the minimum values that induced rupture in all of the attempts made. In our experimental system, these conditions were a pixel dwell time of 32.77 μs and 100 iterations (refer to Step 2 for the acquisition parameters) (Figure 1F).

### 4.2 BAF recruitment at the site of micronuclei and primary nuclei rupture

**FIGURE 2:**
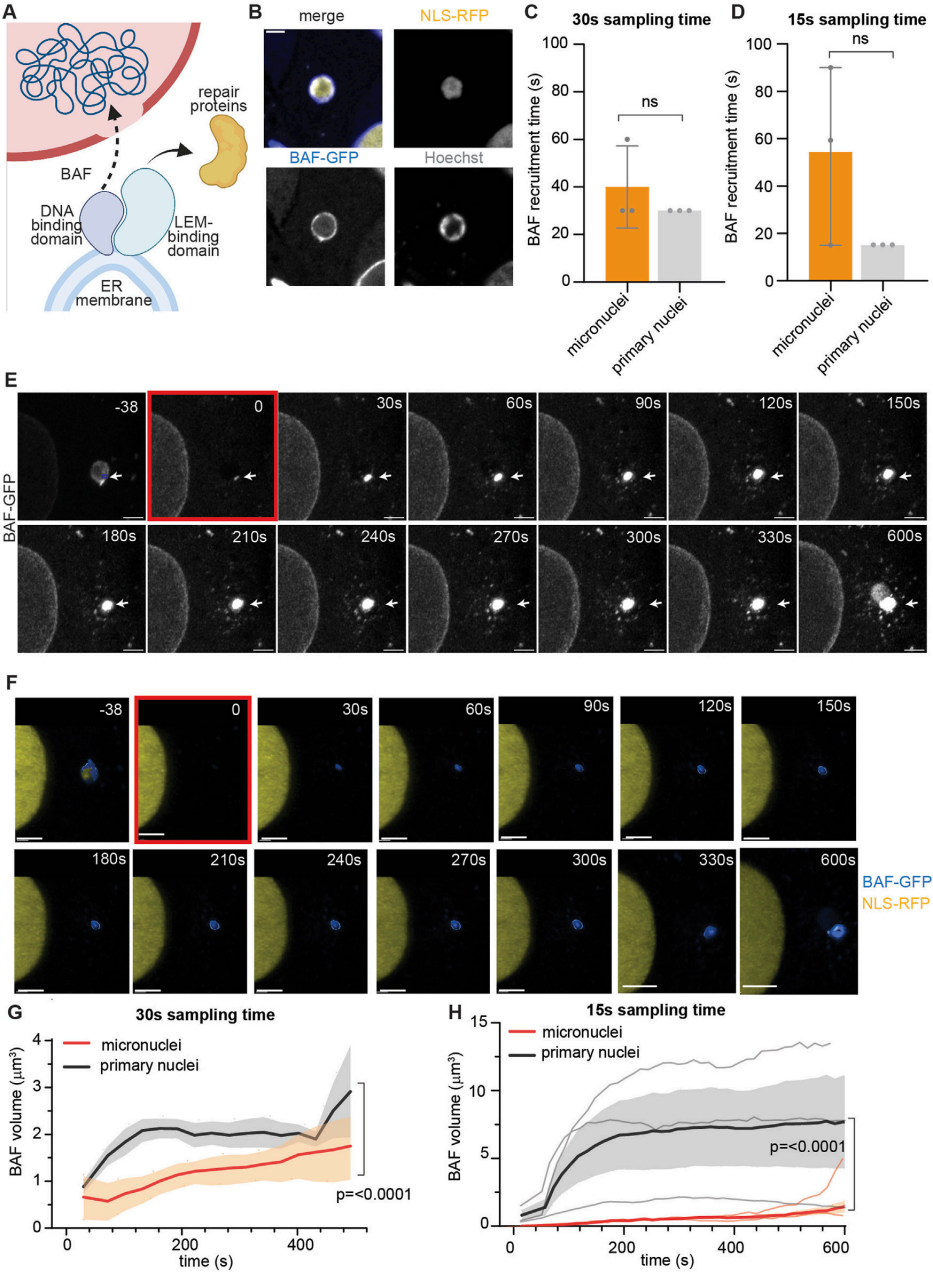
Barrier-to-autointegration factor post-rupture recruitment is impaired at micronuclei. **(A)** Schematic representation of the role of BAF (blue) in nuclear repair. At the site of rupture, BAF is recruited to the exposed chromatin by its DNA-binding domain. This enables repair both by directly bringing the ER membrane to the rupture site to plug the hole and by recruiting other repair proteins through the LEM-binding domain. **(B)** Representative images of HeLa cells expressing NLS-RFP (yellow), BAF-GFP (blue), and stained for Hoechst (gray). Scale bar = 2 μm. **(C)** Calculated BAF arrival time at the rupture site of micronuclei and primary nuclei using a 30 s sampling time. **(D)** Calculated BAF arrival time at the rupture site of micronuclei and primary nuclei using a 15 s sampling time. **(B, C)** For three biological replicates, mean ± standard deviation is plotted. Statistical significance is calculated using unpaired student’s t-test. **(E)** Representative maximum intensity Z-projections of BAF-GFP recruitment at the site of micronuclear rupture. The rupture location is indicated with a white arrow throughout the measurement, while the ROI used for bleaching (blue) is indicated only in the initial frame. In the last frame of the measurement (600 s), the micronucleus shape is visible in the BAF-GFP channel, indicating BAF recruitment to the whole micronucleus. Scale bar = 2 μm. **(F)** Three-dimensional reconstruction of the images shown in **(D)** (overlayed to the fluorescent original image in the background) of HeLa cells expressing BAF-GFP (blue) and NLS-RFP (yellow). The frame in which rupture is induced is highlighted in red. Scale bar = 6 μm. **(G)** Representation of the variation in time of BAF volume in primary nuclei (black) versus micronuclei (red) calculated as in **(E, F)** from measurements taken using a 30-s interval. We ruptured primary nuclei and micronuclei in different cells to avoid proteins being recruited at the same time at two different sites. Nonetheless, the conditions used to rupture primary and micronuclei are the same. Mean ± SEM of three biological replicates, statistical significance was calculated using the Mann–Whitney test, and *p*-value is indicated. **(H)** Representation of the variation in time of BAF volume in primary nuclei (black) versus micronuclei (red), calculated as in **(E, F)** from measurements taken using a 15-s interval. We ruptured primary nuclei and micronuclei in different cells, to avoid proteins being recruited at the same time at two different sites. Nonetheless, the conditions used to rupture primary and micronuclei are the same. Mean ± SEM of three biological replicates, statistical significance was calculated using the Mann–Whitney test, and *p*-value is indicated. Individual curves are overlapped in gray (primary nuclei) and orange (micronuclei).

Next, we used our pipeline to study the recruitment of the primary repair protein BAF at the site of nuclear envelope rupture (Halfmann et al., 2019; Halfmann and Roux, 2021; Young et al., 2020) (Figure 2A). After generating HeLa cells expressing NLS-RFP and the fusion protein BAF-GFP, we used them to visualize BAF dynamics after inducing nuclear rupture (Figure 2B).

Given the irreversibility of the rupture occurring at micronuclei, we wondered whether there was any difference in BAF recruitment at the primary nucleus or at the micronuclear site. To investigate this, we used ImageJ to calculate the timing of BAF recruitment following primary and micronuclear rupture—specifically the velocity of BAF arrival at the rupture site. Figures 2C, D illustrate the impact of reducing the sampling time. We showed that decreasing the sampling time from 30 to 15 s allowed us to capture a more pronounced difference, although not statistically significant, in BAF recruitment time between micronuclei and primary nuclei. This difference was mostly lost using the 30 s sampling time, proving the importance of high temporal resolution in investigating fast processes such as nuclear envelope repair.

We then asked if the extent of BAF recruitment was different at the site of primary or micronuclear rupture. To answer this, we used the software Imaris for 3D volumetric reconstruction (Figures 2E, F; Supplementary Figure S2) and quantified the changes in the volume of BAF after inducing rupture. As shown in Figures 2G, H, the amount of BAF recruited at the primary nuclei is greater than at sites of micronuclear rupture.

### 4.3 CHMP7 recruitment at primary nuclear envelope rupture through LEMD2

**FIGURE 3:**
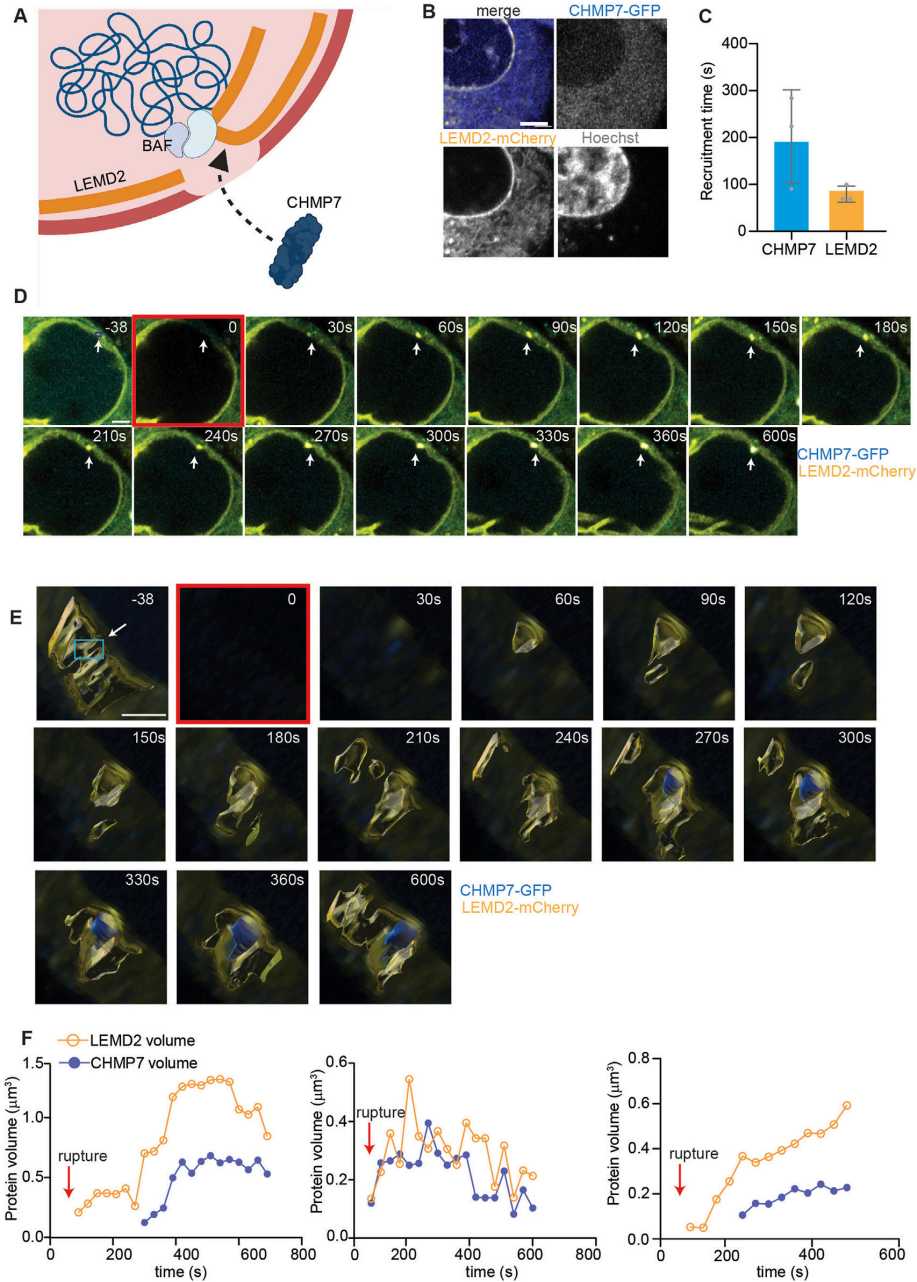
CHMP7 recruitment at sites of primary nuclei rupture shadows LEMD2 dynamics. **(A)** Schematic representation of LEMD2-CHMP7 recruitment at the site of primary nuclei rupture. LEMD2, an inner nuclear membrane protein, is recruited at the rupture site by the BAF LEM-binding domain. CHMP7, a cytosolic protein associated to the endoplasmic reticulum, is recruited at the rupture site through its interaction with LEMD2. **(B)** Representative images of HeLa cells expressing LEMD2-mCherry (yellow), CHMP7-GFP (blue), and stained for Hoechst (gray). Scale bar = 2 μm. **(C)** Calculated arrival time at the rupture site of CHMP7 and LEMD2, showing how the latter is recruited first. Mean ± standard deviation of three biological replicates is plotted. **(D)** Representative maximum intensity Z-projection of HeLa cells expressing CHMP7-GFP (blue) and LEMD2-mCherry (yellow) in which rupture has been induced on primary nuclei. Scale bar = 2 μm. The rupture location is indicated with a white arrow throughout the measurement, while the ROI used for bleaching (blue box) is indicated only in the initial frame. **(E)** Three-dimensional reconstruction of an ROI of 5 μm × 5 μm encompassing the rupture site of images shown in **(D)** of HeLa cells expressing CHMP7-GFP (blue) and LEMD2-mCherry (yellow). The frame in which rupture is induced is highlighted in red. Scale bar = 2 μm. In the initial frame, we indicated the ROI used for bleaching with a white arrow and a blue box. **(F)** Individual curves showing the trend in CHMP7 (blue) and LEMD2 (yellow) volume after primary nuclear rupture, calculated from images as in **(D, E)**. We reconstructed only a narrow region around the rupture location, and we measured and plotted only CHMP7 and LEMD2 independent volumes and not their co-localization. Each graph represents an independent experiment conducted using the same rupture settings, and the rupture event is indicated with the red arrow.

Recognizing the critical role of protein recruitment in the early stages of nuclear envelope repair, we sought to further investigate these processes at primary nuclei with our pipeline. To this aim, we focused on the sequential recruitment of LEM Domain-2 (LEMD2) and charged multivesicular protein 7 (CHMP7) at the site of primary nuclear envelope rupture (Figure 3A). LEMD2-mediated recruitment of the ESCRT-III membrane remodeling complex scaffolding protein, CHMP7, is a crucial step for primary nuclei envelope repair in which it allows for the subsequent nucleation and function of the ESCRT-III complex, which repairs the nuclear envelope (Raab et al., 2016; Gu et al., 2017; von Appen et al., 2020; Thaller et al., 2019). After generating HeLa cells stably expressing CHMP7-GFP and LEMD2-mCherry (Figure 3B), we applied our laser-induced rupture pipeline and studied the behavior of the two proteins after primary nuclei envelope rupture. Despite the variability in CHMP7 arrival time, our method was able to confirm that the recruitment of CHMP7 is secondary to the appearance of LEMD2, as reported in literature (Gu et al., 2017; von Appen et al., 2020; Thaller et al., 2019) (Figure 3C). Then, we asked if a correlation existed between LEMD2 quantity and CHMP7 recruitment extent. Using 3D volume reconstruction, we indeed showed that the trend in CHMP7 volume changes is mirroring LEMD2 quantity variation (Figures 3D–F).

### 4.4 Pitfalls and limitations

Our microscopy pipeline is limited to single-cell studies and cannot be applied to population studies. Moreover, the recruitment of repair proteins at small and localized laser-induced ruptures might not fully represent physiological processes, as different repair mechanisms are usually deployed to address ruptures of different sizes (Halfmann et al., 2019; Young et al., 2020). In addition, we deem the application of these methods to cells in suspension more challenging, due to the problems in focusing and imaging inherently motile cells. Moreover, the overexpression of some envelope proteins such as lamins or, as happened in our experiments, cGAS, might influence the probability of rupture. We thus recommend running a rupture test first, and if the rupture rate is lower than 98%, to recalibrate the iterations or speed of bleaching. Similarly, the laser intensity and pixel size used for imaging may need to be adjusted depending on the expressed proteins and their fluorescent tags. While this should not affect the functionality of the laser-induced rupture step, it is necessary to ensure that the bleaching pixel dwell time and the ROI area remain consistent across different microscopes and experimental conditions.

## 5 Discussion

We developed and optimized a new fluorescence microscopy-based pipeline to study nuclear envelope rupture and the consequent recruitment of repair proteins. Our method efficiently induces rupture in both primary nuclei and micronuclei and distinguishes between the sequence and extent of protein recruitment. By integrating high-resolution microscopy with the established laser-induced rupture, (Halfmann et al., 2019; Denais et al., 2016b; Raab et al., 2016; Kono et al., 2022; Penfield et al., 2018) we achieve precise, controlled rupture both spatially and temporally. This approach enables us to capture protein behavior immediately following the rupture event at the specific location, offering new insights into the dynamics of nuclear repair processes.

As a paradigm for repair processes, we studied the post-rupture dynamics of the first-responder repair protein, BAF. We identified the time and the extent of BAF recruitment at the site of rupture, showing how BAF dynamics evolves over time. By decreasing the sampling time, we observed an increased gap in the BAF recruitment time between primary nuclei and micronuclei. Although this difference is not statistically significant, the clustering of BAF recruitment times for primary nuclei at the shortest sampled value (i.e., the first frame post-rupture) suggests that further reducing the interval between frames might reveal a significantly faster recruitment of BAF at primary nuclei envelope rupture sites compared to micronuclei.

Furthermore, by comparing BAF quantities in primary nuclei and micronuclei, we demonstrated that post-rupture BAF recruitment is diminished in the latter. This reduction is time-dependent and likely specific to the narrow window immediately following rupture. Trends in our data and previous literature (Halfmann et al., 2019; Young et al., 2020) suggest that BAF recruitment eventually reaches the same plateau in both micronuclei and primary nuclei. This early reduction in BAF recruitment might explain the inefficient repair often observed in micronuclei, along with p62-mediated ESCRT-III degradation induced by mitochondrial ROS (Martin et al., 2024; Di Bona et al., 2024).

Finally, we applied the method to study the sequential recruitment of proteins during nuclear envelope damage. In this instance, we used the inner nuclear membrane protein LEMD2 and the ESCRT-III scaffolding protein CHMP7. We showed that LEMD2 is the first to localize at the site of nuclear envelope rupture, followed by CHMP7 at a second stage. This is in agreement with the nuclear repair theories that identify BAF as the initial responder and recruiter of LEM-domain proteins like LEMD2, which then facilitates the subsequent recruitment of CHMP7 and ESCRT-III (Halfmann and Roux, 2021; Gu et al., 2017; Young et al., 2020). Additionally, we found that the quantity of CHMP7 is closely related to the amount of LEMD2, as evidenced by the trends in the volume-over-time curves for these proteins. In this case, we focused solely on the primary nucleus since an additional non-canonical LEMD2-CHMP7 binding that might bias the results has been described at micronuclei (Di Bona et al., 2024).

This method can be used to characterize treatments and inhibitors that might affect nuclear envelope repair proteins, by comparing their mobility or quantity. Similarly, we envisage this method to also be applicable in studying the dynamics of DNA-sensing cytoplasmic proteins, such as cGAS, and to test its separation of function mutant (i.e., DNA binding vs. cyclase). Finally, we believe this technique could provide a platform for testing the effects of various treatments on the probability of rupture. For example, it can be used to assess the propensity of micronuclei or primary nuclei to rupture after shorter laser exposure under conditions affecting lipid stability. In fact, users could replicate the experiments we performed to characterize the method (Figure 1F) and calculate the probability of rupture by varying parameters such as the velocity of bleaching, under control or treated conditions.

In conclusion, our work presents a new pipeline, easily implementable on any confocal microscope, for precisely studying protein behavior immediately after nuclear envelope rupture. This method allowed us to shed light on the crucial but poorly characterized process of micronuclear repair. Moreover, by demonstrating the method’s applicability in studying the sequential recruitment of proteins, we have paved the way for extensive research on primary and micronuclei envelope rupture and repair. This will enhance our understanding of the mechanisms underlying micronuclear irreversible collapse and potentially offer ways to reverse it, with significant implications for the treatment of chromosomally unstable tumors.

## Data Availability

The original contributions presented in the study are included in the article/Supplementary Material; further inquiries can be directed to the corresponding author.

## Supplementary material

Supplementary Figure S1 Assessing micronuclei effective rupture. **(A)** Representative images of micronuclei in HeLa cells expressing NLS-RFP to which laser rupture has been applied. (Top) Micronucleus bleached, but still intact. Despite bleaching, the micronucleus is discernible in the NLS-RFP channel by increasing the contrast. (Bottom) Representative image of an effectively ruptured micronucleus. In this case, despite increasing the contrast, the micronucleus is not visible in the NLS-RFP channel, denoting effective rupture. The ROI used for bleaching (blue box) is indicated by the white arrow in the left image. Yellow arrowheads indicate the location of the micronucleus. Scale bars = 2 μm. **(B)** Schematic representation of cGAS recruitment at ruptured micronuclei. **(C)** Representative images of micronuclei in HeLa cells expressing NLS-RFP to which laser rupture has been applied. Given the slower dynamics of cGAS recruitment, we used 1 min interval between frames. (Top) Micronucleus bleached, but intact, pre and post-rupture, showing NLS-RFP (yellow) and without cGAS recruitment (blue). (Bottom) Ruptured micronucleus, characterized by loss of NLS-RFP (yellow) and by cGAS-GFP recruitment (blue). The ROI used for bleaching (blue box) is indicated by the white arrow in the left image. Yellow arrowheads indicate the location of the micronucleus. Scale bars = 2 μm. **(D)** Identification of cGAS-GFP recruitment at N = 18 micronuclei classified as ruptured or intact using the presence of NLS-RFP, validating the occurrence of the rupture event.

Supplementary Figure S2 BAF recruitment at primary nuclei. **(A)** Representative maximum intensity Z-projection of BAF-GFP recruitment at the site of primary nuclei rupture. The rupture location is indicated with a white arrow throughout the measurement, while the ROI used for bleaching (blue) is indicated only in the initial frame. Scale bar = 2 μm. **(B)** 3D reconstruction of the images shown in **(A)** (overlayed to the fluorescent original image in the background) of HeLa cells expressing BAF-GFP (blue) and NLS-RFP (yellow). The frame in which rupture is induced is highlighted in red. Scale bar = 2 μm.

Supplementary Movie S1 Representative time-lapse of BAF recruitment to the site of primary nuclear rupture. The movie is generated through ImageJ using 2 frames per second after a maximum intensity Z-projection.

Supplementary Movie S2 Representative time-lapse of BAF recruitment to the site of micronuclear rupture. The movie is generated through ImageJ, using 2 frames per second after a maximum intensity Z-projection.
