# Supplementary figures and images for: A new microscopy pipeline for studying the initial stages of nuclear and micronuclear rupture and repair
Source: Front Cell Dev Biol. 2024 Sep 18;12:1475095. doi: 10.3389/fcell.2024.1475095 (PMC11445188; doi:10.3389/fcell.2024.1475095)

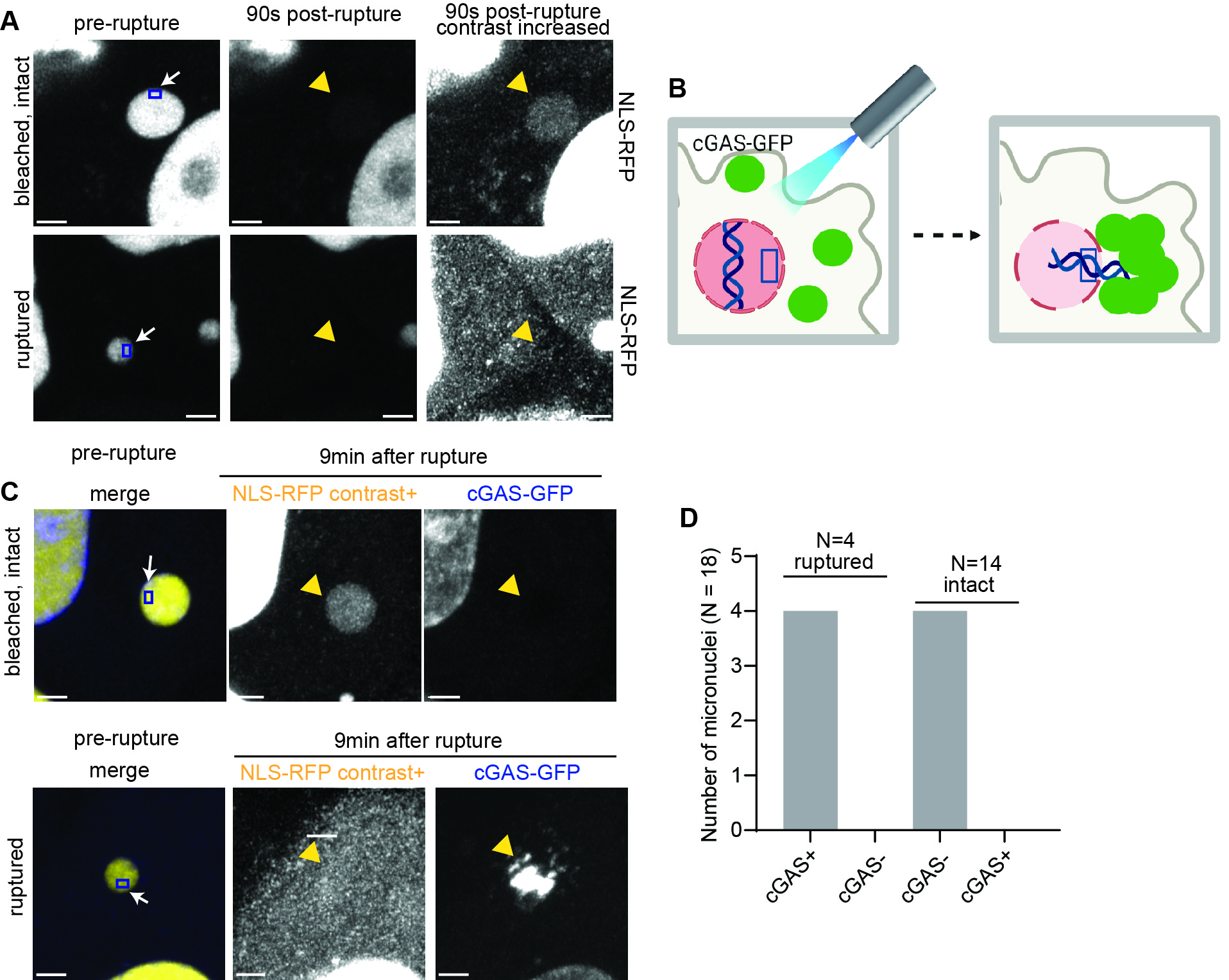

Supplement: Supplementary file 2 [file Image1.JPEG]

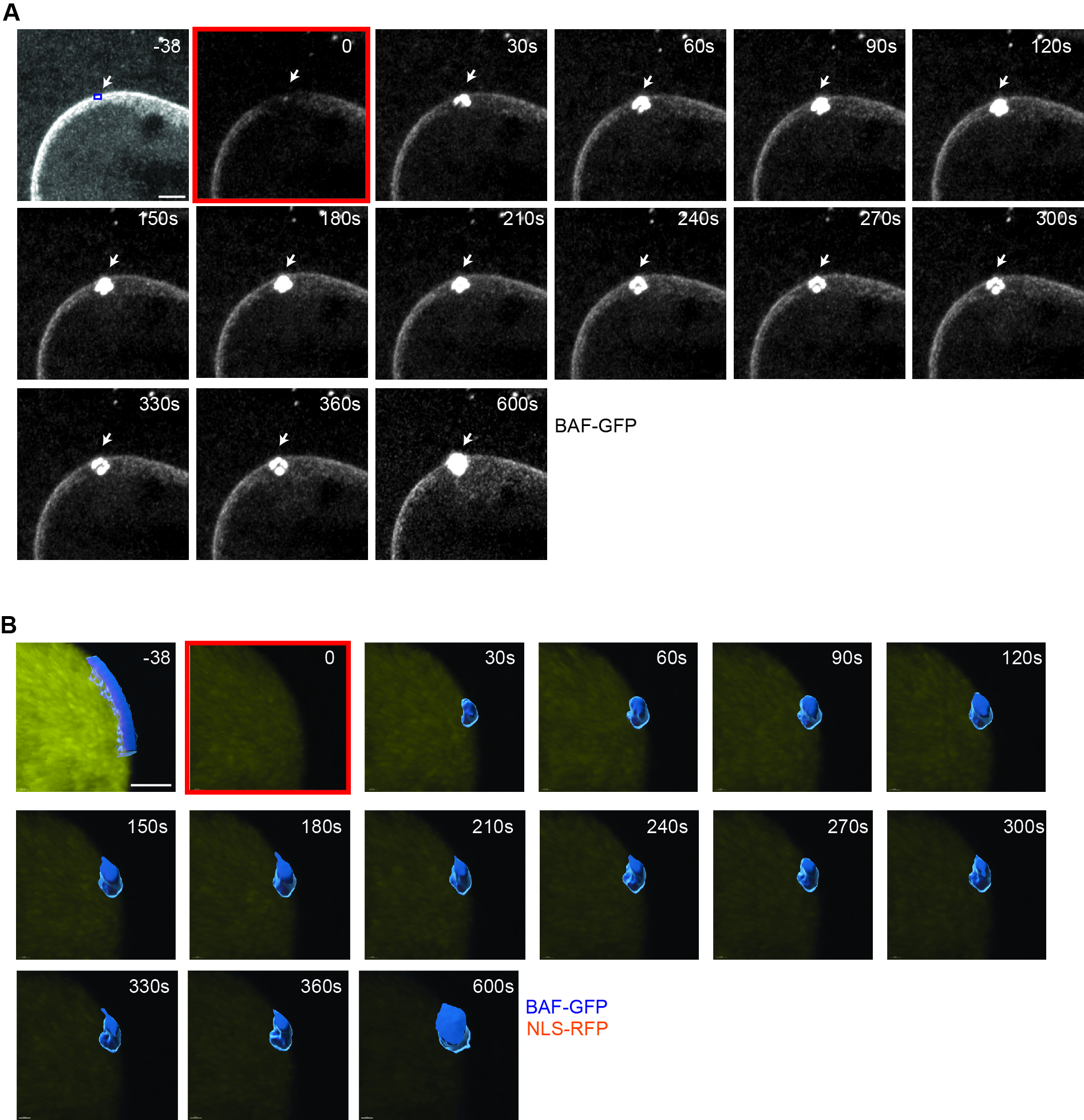

Supplement: Supplementary file 3 [file Image2.JPEG]
